# Perovskites take the lead in local structure analysis

**DOI:** 10.1107/S2052252515024033

**Published:** 2016-01-01

**Authors:** David A. Keen

**Affiliations:** a ISIS Facility, Rutherford Appleton Laboratory, Harwell Oxford, Didcot, Oxfordshire OX11 0QX, United Kingdom

**Keywords:** local structure analysis, pair distribution function analysis, perovskites, single-crystal diffuse scattering

## Abstract

Materials with the much-loved perovskite structure are being used to develop and test new methods for interpreting local structural arrangements in crystals, and these methods are in turn uncovering unexpected new structural insight.

Crystallographers have been drawn to the family of perovskite-structured materials for over 70 years, both because their apparent structural simplicity (Megaw, 1945 ▸) hides a surprisingly rich complexity (Glazer, 1972 ▸), and because the family supports a wealth of important physical properties including, amongst others, (relaxor) ferroelectricity, piezoelectricity, non-linear optics and most recently photovoltaic behaviour (see the focus issue of *Nature Materials* published in September 2014). Indeed the nature of their phase transitions and phase transition sequences are probably some of the most extensively studied of all crystal systems.

Increasingly, however, questions are being asked about the relationship between the average periodic structure, an *ABX*_3_ network of corner-linked *BX*_6_ octahedra, and the local atomic arrangements that make up this structure. Is the structure homogeneous over all length scales and if it isn’t what role does disorder play in the physical properties? Or, equally fundamental, how might local disorder impact on our understanding of phase transitions? Indeed structural inhomogeneity is now known to play a key role in the enhanced piezoelectricity at the morphotropic phase boundary of PZT, PbZr_1− *x*_Ti_*x*_O_3_ (Zhang *et al.*, 2014 ▸) and there is a persistent erosion of the classic displacive picture of ferroelectric phase transitions with the observation of local low-symmetry character in the paraelectric phase of several systems, the latter occurring when specific atoms or molecules are not ‘comfortable’ in high-symmetry positions (Keen & Goodwin, 2015 ▸).

It is well established that the presence of diffuse scattering in a single-crystal diffraction pattern indicates that the structure includes deviations from the average structure. The difficult part is often to identify and quantify the structural origin of the scattering, frequently relying on intuitive model building approaches. Similarly, the pair distribution function (PDF) obtained from careful diffraction experiments from powdered samples is correctly touted as a way to determine local structural arrangements (Farrow *et al.*, 2007 ▸). Occasionally, collating information gleaned from both approaches is required to gain the necessary insight into a single problem. A good example where this was the case is the structure of ferroelectric tetragonal BaTiO_3_ (see Fig. 1 ▸). Qualitative interpretation of the single-crystal diffuse scattering (SCDS) from KNbO_3_ (isostructural to BaTiO_3_) in 1970 (Comès *et al.*, 1970 ▸) led to a local picture of the structure of chains of neighbouring Ti atoms each displaced along one of the four ±1,±1,1 directions to give a macroscopic tetragonal average displacement. Much later analysis of the neutron PDF data confirmed this atomic arrangement and, importantly, quantified the local bonding by revealing the three short, three long Ti–O bond lengths required for the proposed Ti displacements (Levin *et al.*, 2014 ▸).

How to decide whether a single-crystal diffuse scattering measurement or pair distribution function measurement – or indeed a combination of the two – is preferable for a given structural disorder problem is the main topic of an article by Whitfield *et al.* (2016 ▸) in this issue. Here the authors analyse PDFs from the relaxor ferrroelectric perovskite PZN, PbZn_1/3_Nb_2/3_O_3_, in a variety of ways and compare the results with those obtained from Monte Carlo modelling of SCDS. The authors accurately identify and discuss the issues in making this judgement: availability (or not) of single crystals; simplicity of measurement; differences in neutron or X-ray scattering contrast; impact of powder averaging *etc*. They then concentrate on two methods for analysing the PDFs – ‘small box’ and ‘big box’ methods. Again this is a choice that can only be made on a case-by-case basis: small box methods such as *PDFgui* (Farrow *et al.*, 2007 ▸) are quick and straightforward but for systems where the disorder extends beyond the unit cell the results can be somewhat limited; large box methods [*e.g.* reverse Monte Carlo (RMC) refinements (Tucker *et al.*, 2007 ▸)] are more time-consuming but are more generally applicable.

When assessing the big box methods the authors do not use the routinely available RMC codes such as *RMCprofile* (Tucker *et al.*, 2007 ▸) that also fit reciprocal space data – especially the Bragg powder profile – and so their comparisons to SCDS are somewhat compromised. This is a missed opportunity as it is known that without restraining the average structure PDF-based big box models can lose long-range coherence (Levin *et al.*, 2014 ▸). Notwithstanding this the authors’ approach highlights a further important point: methods such as RMC work best as refinements of starting structures and refinements work best with the best quality data.

There is now a wealth of evidence to show that the more local structure is investigated the more we are obliged to reassess our understanding of crystalline structure and behaviour and, perhaps typically, perovskite-structured systems are again at the forefront (*e.g.* Keeble *et al.*, 2013 ▸, Senn *et al.*, 2015 ▸). The article by Whitfield *et al.* (2016 ▸) emphasizes the point that average structures are just that and, as materials’ properties often depend on the local structure, it is important to understand of what they are averages. Developing, optimizing and critically assessing the tools that we have available to uncover local structure in crystals is now a vital part of crystallography.

## Figures and Tables

**Figure 1:**
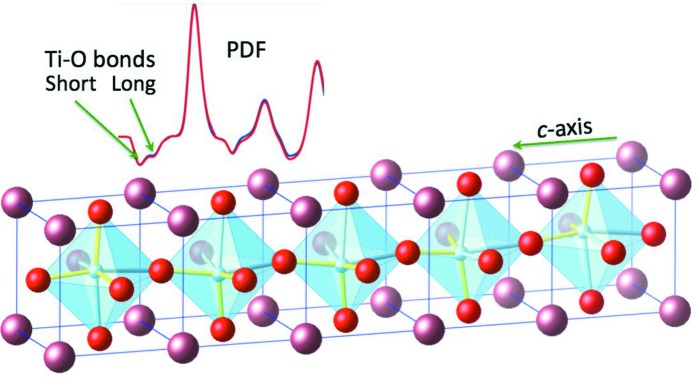
Schematic representation of the local arrangement of Ti atoms in the tetragonal perovskite BaTiO_3_ based on the single-crystal diffuse scattering work of Comès *et al.* (1970 ▸) and RMC analysis of neutron PDF and electron diffraction data in Levin *et al.* (2014 ▸). Blue Ti atoms within the TiO_6_ octahedra are displaced in 111 directions to produce three short (yellow) and three long (blue) Ti–O bonds.
